# Herbal Extract-Induced DNA Damage, Apoptosis, and Antioxidant Effects of *C. elegans*: A Comparative Study of *Mentha longifolia*, *Scrophularia orientalis*, and *Echium biebersteinii*

**DOI:** 10.3390/ph18071030

**Published:** 2025-07-11

**Authors:** Anna Hu, Qinghao Meng, Robert P. Borris, Hyun-Min Kim

**Affiliations:** 1 Division of Natural and Applied Sciences, Duke Kunshan University, Kunshan 215316, China; 2 School of Pharmaceutical Science and Technology, Tianjin University, Tianjin 300072, China

**Keywords:** *Mentha longifolia*, *Scrophularia orientalis*, *Echium biebersteinii*, DNA repair, meiosis, germline development, medicinal plants

## Abstract

**Background:** Herbal medicine represents a rich yet complex source of bioactive compounds, offering both therapeutic potential and toxicological risks. **Methods:** In this study, we systematically evaluated the biological effects of three traditional herbal extracts—*Mentha longifolia*, *Scrophularia orientalis*, and *Echium biebersteinii*—using *Caenorhabditis elegans* as an in vivo model. **Results:** All three extracts significantly reduced worm survival, induced larval arrest, and triggered a high incidence of males (HIM) phenotypes, indicative of mitotic failure and meiotic chromosome missegregation. Detailed analysis of germline architecture revealed extract-specific abnormalities, including nuclear disorganization, ectopic crescent-shaped nuclei, altered meiotic progression, and reduced bivalent formation. These defects were accompanied by activation of the DNA damage response, as evidenced by upregulation of checkpoint genes (*atm-1*, *atl-1*), increased pCHK-1 foci, and elevated germline apoptosis. LC-MS profiling identified 21 major compounds across the extracts, with four compounds—thymol, carvyl acetate, luteolin-7-*O*-rutinoside, and menthyl acetate—shared by all three herbs. Among them, thymol and carvyl acetate significantly upregulated DNA damage checkpoint genes and promoted apoptosis, whereas thymol and luteolin-7-*O*-rutinoside contributed to antioxidant activity. Notably, *S. orientalis* and *E. biebersteinii* shared 11 of 14 major constituents (79%), correlating with their similar phenotypic outcomes, while *M. longifolia* exhibited a more distinct chemical profile, possessing seven unique compounds. **Conclusions:** These findings highlight the complex biological effects of traditional herbal extracts, demonstrating that both beneficial and harmful outcomes can arise from specific phytochemicals within a mixture. By deconstructing these extracts into their active components, such as thymol, carvyl acetate, and luteolin-7-*O*-rutinoside, we gain critical insight into the mechanisms driving reproductive toxicity and antioxidant activity. This approach underscores the importance of component-level analysis for accurately assessing the therapeutic value and safety profile of medicinal plants, particularly those used in foods and dietary supplements.

## 1. Introduction

**Figure 1:**
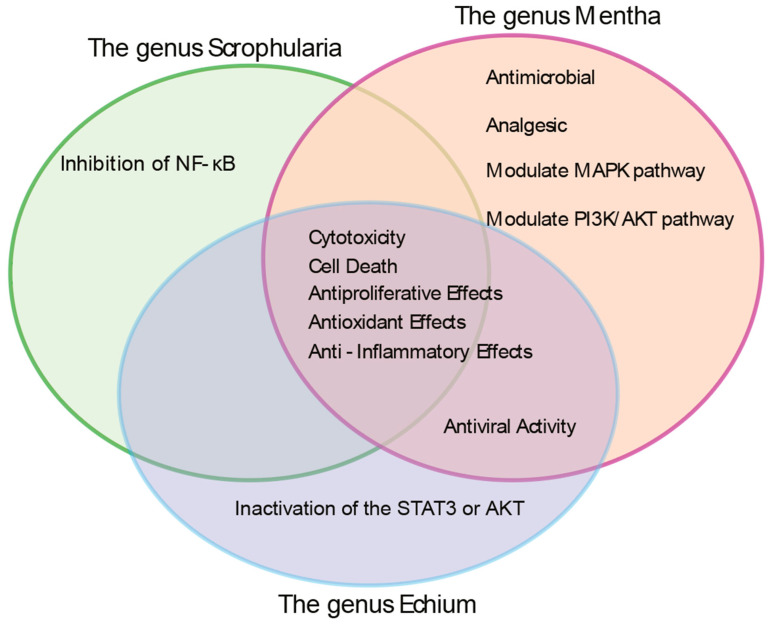
Venn diagram summarizing the reported biological activities of the genera Scrophularia, Mentha, and Echium based on the published literature. This diagram emphasizes the shared biological properties among the three genera from which the herb extracts were derived. Notably, all three have been consistently reported to exhibit antioxidant, pro-apoptotic, anti-inflammatory, cytotoxic, anti-proliferative, and antimicrobial activities. Non-overlapping regions represent additional, genus-specific effects reported in the literature. Detailed information can be found in Table 1, Supplementary Data S1 and S2.

While medicinal plants have long been used in traditional remedies, their molecular effects—particularly on genome stability and reproductive health—remain poorly understood. This gap is especially significant given the rising use of herbal formulations with uncharacterized toxicological profiles. To address this gap, we investigated three herbal species native to Armenia—*Scrophularia orientalis*, *Mentha longifolia*, and *Echium biebersteinii*. All three are known for their strong bioactivity (Figure 1 and Table 1), but their potential roles in DNA damage repair and apoptosis have not been fully validated. We hypothesized that extracts from these herbs may interfere with DNA repair pathways and germline development due to their bioactive components.

### 1.1. Scrophularia orientalis *L.*

The *Scrophularia* genus (Scrophulariaceae) includes 200–300 species across temperate Asia, Europe, and North America [5,7]. These plants feature quadrangular stems, opposite leaves, and globose to subconical capsules with small seeds [6].

*Scrophularia* species are known for their antioxidant, anti-inflammatory, and anticancer effects [5,10,11]. *S. orientalis* extract reduces neuroblastoma cell viability [12]. Other species, such as *S. striata, S. floribunda,* and *S. lucida*, also show anti-proliferative effects on cancer cells [13,14].

### 1.2. Echium biebersteinii Laicata

*Echium* (Boraginaceae) includes ~60 species native to North Africa, Europe, and the Macaronesian islands [8,9,15]. Although *E. biebersteinii* has not been extensively studied, other species in the genus, notably *E. amoenum*, have received considerable pharmacological attention.

Various Echium species exhibit sedative, antioxidant, and anxiolytic effects and are traditionally used to treat respiratory issues, ulcers, and mental disorders [9,16,17,18,19,20]. *E. italicum*, closely related to *E. biebersteinii*, is used in Turkey for wound healing and rheumatic pain [9,21]. *E. amoenum*, the best studied, shows anticancer effects via rosmarinic acid-mediated inhibition of STAT3, AKT, and ERK1/2 [22].

### 1.3. Mentha longifolia (*L.*) *L.*

The genus *Mentha* (mint; Lamiaceae) includes between 18 and 30 species. *M. longifolia* (L.) L. is an aromatic perennial herb widely distributed across Northern Pakistan, Europe, Nepal, India, Western China, Germany, the United Kingdom, Egypt, Nigeria, and Turkey [1,2]. Like other *Mentha* species, it has square stems, aromatic leaves, and spreading stolons [23,24].

Traditionally used for gastrointestinal, respiratory, and inflammatory conditions, *M. longifolia* contains essential oils like menthol with antimicrobial and antifungal effects [25]. Its flavonoids may have anti-HIV activity [26], and extracts show antioxidant and anti-proliferative effects on cancer cells [27,28].

Taken together, the *Scrophularia*, *Echium*, and *Mentha* species highlight the broad pharmacological potential across diverse plant families. Despite phylogenetic differences, they share antioxidant, anti-inflammatory, cytotoxic, and antimicrobial activities, suggesting that common molecular mechanisms may underlie the observed phenotypes.

To explore this hypothesis, we investigated the biological activity of the three herbal species using the *Caenorhabditis elegans* (*C. elegans*) model system. This model provides a powerful in vivo platform for studying the effects of bioactive compounds on development, reproduction, and genomic stability.

Plant-specific solvents were used to prepare the following extracts: *E. biebersteinii* with butanol, *M. longifolia* with dichloromethane, and *S. orientalis* with water. All extracts were subsequently resuspended in a standardized DMSO–water mixture to ensure consistency in treatment conditions.

Comparative analysis revealed that exposure to each of the three herbal extracts significantly reduced worm survival compared to untreated controls. Treated worms exhibited larval arrest or lethality, suggesting that impaired survival may be linked to disruptions in mitotic cell division during larval development. Notably, all three extracts induced a high incidence of males progeny (HIM phenotype), implying disruption of sex chromosome segregation and potential interference with meiotic processes.

Further analysis revealed a reduced number of DAPI-stained bodies and abnormal meiotic progression in the germline of treated worms, providing additional evidence for impaired meiotic development. Consistently, treatment with any of the three extracts activated the DNA damage checkpoint response via the ATM/ATR and CHK-1 pathways. This response was accompanied by defective germline development, indicating that the extracts interfere with DNA damage repair mechanisms and ultimately lead to fertility defects.

To elucidate the molecular basis of these phenotypes, we performed LC-MS analysis of the herbal extracts. Several shared components—luteolin-7-*O*-rutinoside, thymol, carvyl acetate, and menthyl acetate—were identified, each having been previously associated with oxidative stress regulation, apoptosis induction, or genotoxic effects. These compounds are likely contributors to the observed disruptions in worm development and reproduction.

Interestingly, *S. orientalis* and *E. biebersteinii* shared 79% of their major compounds, indicating a high degree of chemical similarity. In contrast, *M. longifolia* shared only 42% of its compounds with the other two and possessed seven unique compounds (58%), reflecting a more distinct chemical profile. These differences may underlie the variable biological responses observed in the *C. elegans* assays and suggest plant-specific mechanisms of action.

This study reveals that extracts from *M. longifolia*, *S. orientalis*, and *E*. *biebersteinii* induce reproductive defects in *C. elegans* by activating DNA damage checkpoints and apoptotic pathways. High-resolution imaging of germline architecture linked structural abnormalities—such as disorganized nuclei, impaired meiotic progression, and reduced bivalent formation—to molecular stress responses. All three extracts significantly decreased survival, caused larval arrest, and increased the high incidence of the male (HIM) phenotype, indicating chromosomal missegregation.

We identified 21 major compounds, including four shared across the extracts. Among them, thymol and carvyl acetate were associated with pro-apoptotic activity, while thymol and luteolin-7-*O*-rutinoside exhibited antioxidant effects. These findings highlight both conserved and compound-specific mechanisms of herbal reproductive toxicity and support the use of *C. elegans* as a model for functional toxicological screening of traditional remedies. Also, this study underscores the need to analyze individual phytochemicals within herbal mixtures to understand their distinct biological effects.

## 2. Results

**Figure 2:**
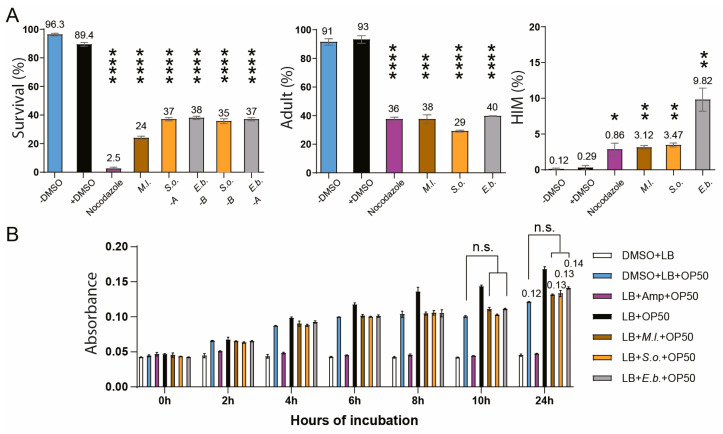
Extracts obtained from *M. longifolia* (*M.l.*), *S*. *orientalis* (*S.o.*), and *E*. *biebersteinii* (*E.b.*) exhibit marked nematocidal, larval arrest/lethality, and HIM phenotype of *C. elegans*, without exerting discernible impact on bacterial growth. (**A**) *M.l.*, *S.o.*, and *E.b*. extracts significantly diminished survival and larval development while augmenting the high incidence of males (HIM) phenotype in *C. elegans*. The effect of herb extracts was evaluated by treating worms with different extracts of *M.l.*, *S.o.*, and *E.b.* and monitoring their survival, adult formation, and male (HIM) phenotype over a 48 h period. Statistical significance was assessed using a two-tailed *t*-test, with * *p* < 0.05; ** *p* < 0.01; *** *p* < 0.001; and **** *p* < 0.0001, comparing the control (+DMSO) with the treated samples. Nocodazole is a positive control. (**B**) Assessment of bacterial growth in the presence of herbal extracts. *E. coli* OP50 was incubated with 0.03 μg/mL of *M.l.*, *S.o.*, and *E.b.* extracts—the same concentration used in *C. elegans* assays—for 24 h. No significant inhibition of bacterial growth was observed at absorbance (600nm), indicating that the extracts’ nematocidal effects are unlikely to result from compromised bacterial food source (*p* = 0.100 for all three herbs at 24 h of incubation).

All three plant extracts exhibited potent nematocidal activity after 48 h of treatment at 20 °C, with survival rates ranging from 24% to 38%, compared to 89.4% in the DMSO-treated control group (Figure 2A). In addition to reduced survivability, extract-treated worms exhibited a larval arrest or lethality (93% vs. 38% for DMSO and *M.l.*, *p* = 0.0002; 93% vs. 29% for *S.o.*, *p* < 0.0001; 93% vs. 40% for *E.b.*, *p* < 0.0001; two-tailed *t*-test), suggesting that decreased viability is likely linked to mitotic growth defects.

Also, all three extracts significantly increased the incidence of the high incidence of males (HIM) phenotype, indicative of potential sex chromosome missegregation and aberrant meiotic development (0.29% vs. 3.12% for DMSO and *M.l.*, *p* = 0.002; 3.47% for *S.o.*, *p* = 0.0015; 9.82% for *E.b.*, *p* = 0.0044; two-tailed *t*-test; [29]).

To explore whether the choice of initial extraction solvent might influence the biological activity of the plant extracts, we compared extracts prepared using different solvents in selected cases. In *S. orientalis*, extracts obtained with water (*S.o*-A) and butanol (*S.o*-B) showed similar survival-promoting effects (Figure 2A, 37% and 35% survival, respectively). Likewise, *E. biebersteinii* extracts prepared with butanol (*E.b*-B) and water (*E.b*-A) yielded comparable survival rates (38% vs. 37%) and produced similar phenotypic outcomes. These observations suggest that while different solvents may extract distinct chemical components, their impact on functional outcomes such as survival and stress resistance may be limited in some cases.

Since *C. elegans* feeds on *E. coli*, we tested whether the observed nematocidal effects might result from indirect toxicity due to impaired bacterial growth. However, bacterial growth curves showed no significant changes following treatment with any of the three extracts at 0.03 μg/mL—the same concentration that induced phenotypes in *C. elegans*—indicating minimal impact on bacterial proliferation (Figure 2B). After 24 h of incubation, the OD600 values were comparable across groups: 0.12 for DMSO + *E. coli*, 0.13 with *M.l*., 0.13 with *S.o*., and 0.14 with *E.b*.

**Figure 3:**
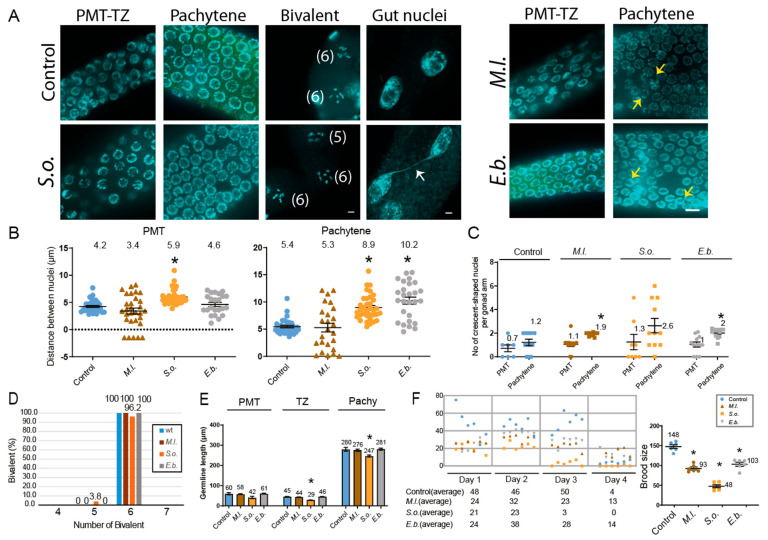
Effects of herbal extracts on nuclear organization, germline development, and fertility-defective outcomes in *C. elegans*. *S.o*. and *E.b*. herb extracts induced increased spacing between nuclei within the pachytene region. In contrast, the *M.l*. extract did not produce any discernible changes in nuclear spacing or organization. (**A**) DAPI-stained nuclei during germline development of 24 h post-L4 hermaphrodite with or without treatment of three herb extracts. Yellow Arrows indicate crescent-shaped nuclei positioned at pachytene. White arrow indicates chromatin bridge. Worms exposed to the herbal extracts often exhibited a reduced number of DAPI-stained bivalent bodies during diakinesis, with a count of 5 indicating five bivalents and a count of 6 indicating six bivalents. Bar = 2 µm. (**B**) Quantification of the increased nuclear spacing in the PMT and pachytene stages shown in the panel. (**C**) Quantification of crescent-shaped nuclei in per gonad arm is indicated. Asterisks indicate statistically significant differences compared to the control group. (**D**) Quantification of DAPI-stained bivalents in the germline. The percentage of bivalents at −1 position of the oocyte is indicated. Numbers in the brackets in panel A indicate the number of bivalents. (**E**) Germline length was measured in three regions: the PMT, TZ, and pachytene. *S.o*. extract shortens specific TZ and pachytene stage. (**F**) Brood size of herb-exposed hermaphrodites. The number of offspring produced by individual hermaphrodite worms was monitored daily over a four-day reproductive period following treatment with herbal extracts. Data are presented as mean ± SEM. Statistical significance was assessed using a two-tailed *t*-test. Asterisks indicate statistically significant differences compared to the control group.

In *C. elegans*, germline nuclei are organized in a well-defined spatial and temporal pattern during germline development. Actively dividing mitotic nuclei are located at the distal end within the premeiotic tip (PMT), and as cells move proximally, they enter meiotic prophase, beginning at the transition zone (TZ), where nuclei display a characteristic crescent-shaped morphology [29]. To assess effects on germline architecture, adult hermaphrodites were dissected, DAPI-stained, and analyzed. In controls, germline nuclei maintained orderly progression from the premeiotic tip (PMT) through the transition zone (TZ) to the pachytene region (Figure 3A). However, *S.o.* and *E.b.* treatments caused increased nuclear gaps, especially in the pachytene region, while *S.o.* additionally affected the PMT. In contrast, *M.l.* had no visible impact on nuclear organization (Figure 3B).

Crescent-shaped nuclei, normally restricted to the transition zone (TZ) in controls, appeared ectopically in both the pre-meiotic tip (PMT) and pachytene regions of extract-treated worms (Figure 3C). While control animals showed proper localization of these nuclei to the TZ, all three herbal extracts induced their mislocalization into adjacent germline regions. This mislocalization increased significantly: *M.l.* (1.2 vs. 1.9, 1.58-fold, *p* = 0.0103), *S.o.* (1.2 vs. 2.6, 2.17-fold, *p* = 0.0649), and *E.b.* (1.2 vs. 2.0, 1.67-fold, *p* = 0.0088). These findings suggest premature entry into meiosis and disrupted developmental timing.

At the diakinesis stage, control worms showed the expected six DAPI-stained bivalents, whereas *S.o.*-treated animals showed five bivalents in 3.8% of cases, indicating potential homologous recombination or synapsis defects (Figure 3A,D; [30]). No abnormal bivalent numbers were detected in *M.l.* or *E.b.* groups.

Proper spatial organization of germline nuclei reflects normal developmental progression, and its disruption is often associated with reduced germline size. A significant decrease in germline length was observed only in worms treated with *S. orientalis* extract. The TZ and pachytene region lengths decreased from 45 μm to 29 μm (Figure 3E, *p* = 0.0006) and from 280 μm to 247 μm (*p* = 0.0175), respectively. No significant changes were observed in the PMT length (60 μm vs. 42 μm, *p* = 0.0519).

These developmental defects correlated with reduced fertility, as evidenced by a decrease in brood size over four days. The most notable reduction occurred on day 3. *S.o.*-treated worms showed a 3.08-fold decline in brood size (Figure 3F, 148 to 48, *p* = 0.0022), while *M.l.* and *E.b.* led to 1.59-fold (to 93, *p* = 0.0022) and 1.44-fold (to 103, *p* = 0.0050) reductions, respectively. These results suggest that impaired germline development ultimately leads to reduced fertility.

**Figure 4:**
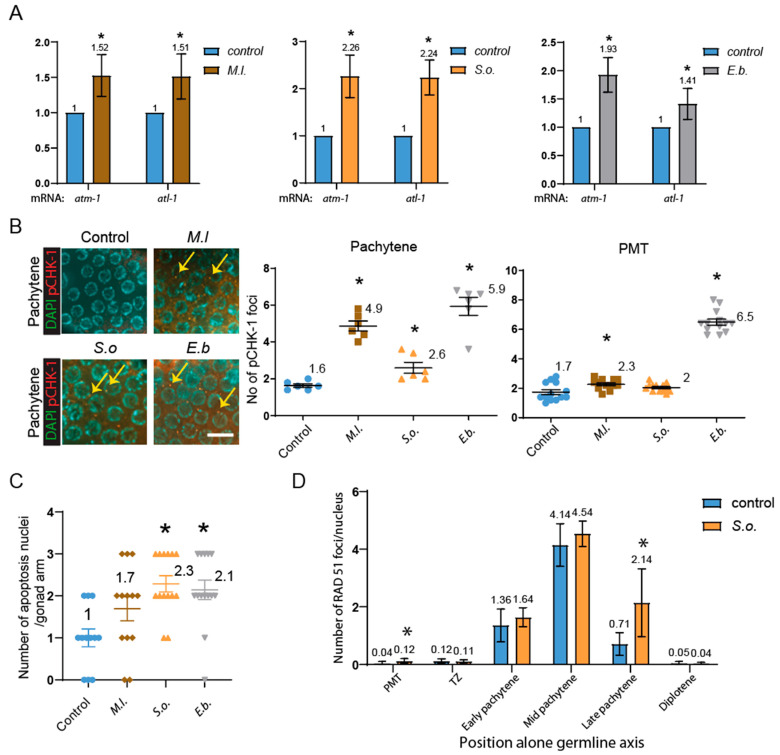
The three-herb extract exposure activates the DNA damage checkpoint pathway and apoptosis. *S.o*. extract leads to defective DSB repair in the germline. (**A**) Quantitative PCR analysis of DNA damage checkpoint gene expression in whole worms treated with herbal extracts. Transcript levels of *atm-1* and *atl-1* were normalized to *tba-1* (tubulin) and compared to untreated controls. (**B**) Quantification of pCHK-1 foci, a downstream marker of ATM/ATR checkpoint activation, in the premeiotic tip (PMT) and pachytene region. All three herb treatments significantly increased pCHK-1 foci in the pachytene stage. Arrows indicate pCHK-1 foci adjacent to chromatin. Bar = 2 µm. (**C**) Quantification of germline apoptosis using acridine orange staining. Apoptotic nuclei were significantly elevated in the pachytene region following *S.o*. and *E.b*. treatments, while *M.l*. treatment caused a mild, non-significant increase compared to the control. (**D**) RAD-51 foci quantification to assess double-strand break (DSB) repair. *S.o*. treatment led to significantly increased RAD-51 foci in both the PMT and late pachytene, indicating impaired DSB repair during both mitotic and meiotic stages. All statistical analyses were performed using two-tailed Mann–Whitney tests. Data are presented as mean ± SEM from biological replicates. Asterisks indicate statistically significant differences compared to the control group.

We hypothesized that impaired germline progression would activate the DNA damage checkpoint and initiate DNA repair mechanisms. To determine whether germline disruption was associated with activation of the DNA damage response, we assessed expression of DNA damage checkpoint genes. All three extracts significantly upregulated *atm-1* and *atl-1* mRNA, two key DNA damage checkpoint kinases: *M.l.* (Figure 4A, 1.52- and 1.51-fold), *S.o.* (2.26- and 2.24-fold), and *E.b.* (1.93- and 1.41-fold); *p* = 0.0007 for all (Mann–Whitney test).

Consistent with the upregulation of key DNA damage checkpoint genes, an increase in pCHK-1 foci was observed in the pachytene region following treatment with *M.l.* (1.6 vs. 4.9, *p* = 0.0049), *S.o.* (2.6, *p* = 0.0076), and *E.b.* (5.9, *p* = 0.0049) (Figure 4B). Additional pCHK-1 foci appeared in the PMT for *M.l.* (1.7 vs. 2.3, *p* = 0.0263) and *E.b.* (1.7 vs. 6.5, *p* < 0.0001), but not significantly for *S.o.* (1.7 vs. 2.0, *p* = 0.0981).

Activation of the DNA damage checkpoint along with meiotic defects would lead to DNA damage-mediated cell death in the pachytene stage of the germline in *C. elegans*. In line with this idea, apoptosis in the pachytene region increased significantly in *S.o.* (Figure 4C, 1 vs. 2.3, p = 0.0008) and *E.b.* (1 vs. 2.1, *p* = 0.0024)-treated groups. *M.l.* induced a mild, non-significant increase (1 vs. 1.7, *p* = 0.0776). This apoptotic response was especially pronounced in worms treated with *S. orientalis* and *E. biebersteinii*, underscoring their stronger detrimental effects on germline integrity.

Among the three, *S.o.* induced the most pronounced phenotypes—altered nuclear organization, reduced bivalents, shortened germline regions, decreased brood size, and elevated expression of DNA damage markers—prompting further analysis of DNA repair. To further investigate this, we analyzed RAD-51 foci, which mark sites of double-strand break (DSB) repair [31,32]. RAD-51 foci were significantly increased in *S.o.*-treated worms at both the PMT (Figure 4D, 0.04 vs. 0.12, *p* = 0.023) and late pachytene stages (0.71 vs. 2.14, *p* = 0.0028), suggesting impaired double-strand break (DSB) repair (Figure 4D). Although RAD-51 foci levels were mildly increased in the transition zone, early pachytene, mid pachytene, and diplotene stages (0.12 vs. 0.11 in TZ, *p* = 0.7430; 1.36 vs. 1.64 in early pachytene, *p* = 0.1443; 4.14 vs. 4.54 in mid pachytene, *p* = 0.0752; 0.05 vs. 0.04 in diplotene, *p* = 0.7317); these differences were not statistically significant.

**Figure 5:**
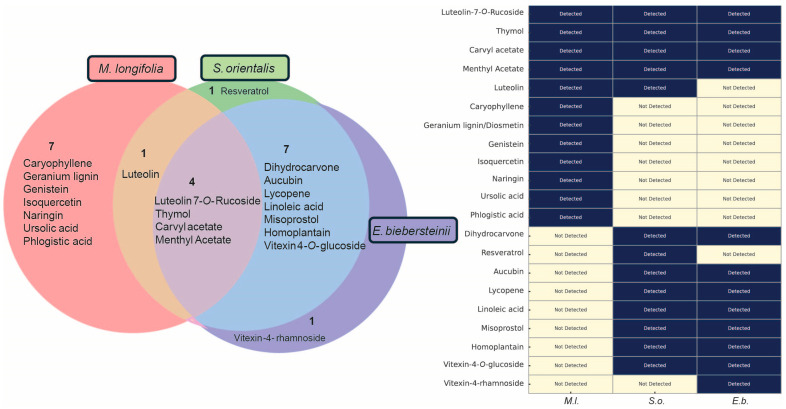
Comparative LC-MS analysis of major compounds in three herbal extracts. Venn diagram and heat map summarizing the 21 major compounds identified across *M*. *longifolia*, *S*. *orientalis*, and *E*. *biebersteinii*. Four compounds—luteolin-7-*O*-rutinoside, thymol, carvyl acetate, and menthyl acetate—were common to all three extracts. *M. longifolia* contained seven unique major compounds. *S. orientalis* had one unique compound, resveratrol, while *E. biebersteinii* uniquely possessed vitexin-4′-rhamnoside. Compound identification was performed based on methods previously described (see Materials and Methods [33,34,35]).

To explore the molecular basis of the distinct phenotypic effects observed in *C. elegans*, we conducted LC-MS analysis on each of the three herbal extracts, as detailed in our previous reports [33,34,35]. This analysis identified 21 major compounds across the extracts (Figure 5), with four compounds—luteolin-7-*O*-rutinoside, thymol, carvyl acetate, and menthyl acetate—common to all. *M*. *longifolia* contained the highest number of unique compounds, including caryophyllene, genistein, and ursolic acid, totaling seven unique constituents. *S*. *orientalis* featured one exclusive compound, resveratrol, while *E*. *biebersteinii* uniquely contained vitexin-4′-rhamnoside. These findings highlight both common and unique chemical profiles that may explain the distinct biological activities of the extracts (Table 2).

Since all three herbs produced common phenotypes—upregulation of DNA damage checkpoint regulators and elevated germline apoptosis—we next investigated whether the four shared compounds could contribute to these effects. Specifically, we examined the expression levels of key DNA damage checkpoint genes following treatment with each compound.

**Figure 6:**
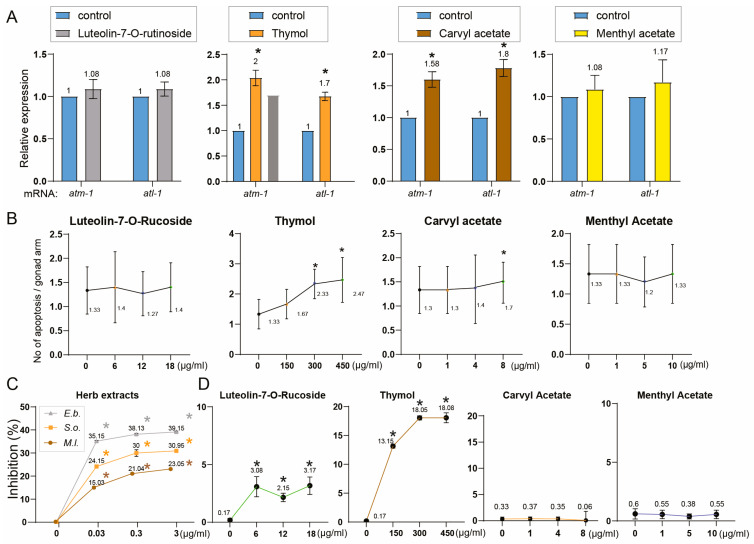
Functional characterization of shared herbal compounds reveals their roles in DNA damage response, apoptosis, and antioxidant activity. (**A**) Expression levels of DNA damage checkpoint genes (*atm-1* and *atl-1*) in response to treatment with four common herbal compounds. Young adult hermaphrodites were treated with luteolin-7-*O*-rutinoside (18 µg/mL), thymol (450 µg/mL), carvyl acetate (8 µg/mL), or menthyl acetate (10 µg/mL), and qRT-PCR was performed to assess expression of *atm-1* and *atl-1*. Thymol and carvyl acetate significantly upregulated both genes, whereas luteolin-7-*O*-rutinoside and menthyl acetate showed no significant effect. Data are presented as fold change relative to control (mean ± SEM, n ≥ 20 animals per group). (**B**) Quantification of germline apoptosis in the pachytene region following compound treatment. Germline apoptosis was measured in wild-type *C. elegans* treated with the four shared compounds. Thymol and carvyl acetate significantly increased apoptotic cell counts, consistent with their induction of *atm-1* and *atl-1* checkpoint gene expression. Luteolin-7-*O*-rutinoside and menthyl acetate showed no significant effects. Data are presented as mean apoptotic nuclei per gonad arm. Mean ± SEM, n ≥ 20 animals per group. (**C**) DPPH radical scavenging activity of *M.l*., *S.o*., and *E.b*. herb extracts at increasing concentrations. All three extracts exhibited dose-dependent antioxidant activity, with *E.b*. showing the strongest inhibition (39.15% inhibition at 3 µg/mL, *p* < 0.0001), followed by *S.o*. (30.95%, *p* < 0.0001) and *M.l*. (23.05%, *p* < 0.0001). (**D**) Antioxidant activity of four common constituents found in the herb extracts at three different doses: luteolin-7-*O*-rutinoside (6–18 µg/mL), thymol (150–450 µg/mL), carvyl acetate (1–8 µg/mL), and menthyl acetate (1–10 µg/mL). Luteolin-7-*O*-rutinoside and thymol showed dose-dependent radical scavenging activity, with thymol demonstrating stronger inhibition (up to 18.08%). In contrast, carvyl acetate and menthyl acetate showed negligible activity at all tested concentrations. Data are presented as mean ± SEM. Statistical significance was calculated using two-tailed Mann–Whitney test. Asterisks indicate statistically significant differences compared to the control group.

Thymol and carvyl acetate significantly upregulated *atm-1* and *atl-1* (Figure 6A, thymol: 2.0- and 1.7-fold; carvyl acetate: 1.58- and 1.8-fold; *p* = 0.0005 for all), whereas luteolin-7-*O*-rutinoside and menthyl acetate had no significant effect (luteolin-7-*O*-rutinoside: *p* = 0.5396 for *atm-1*, *p* = 0.1870 for *atl-1*; menthyl acetate: *p* = 0.6029 for *atm-1*, *p* = 0.1459 for *atl-1*).

To determine whether these compounds also influence germline apoptosis, we quantified DNA damage-induced apoptosis. Thymol and carvyl acetate promoted germline apoptosis (Figure 6B, thymol: 1.33 to 2.47, *p* < 0.0001; carvyl acetate: 1.3 to 1.7, *p* = 0.0359). In contrast, luteolin-7-*O*-rutinoside induced only a marginal, non-significant change (1.05-fold, *p* = 0.7281), and menthyl acetate showed no effect (*p* = 0.9797). Thus, thymol and carvyl acetate may mediate the pro-apoptotic effects of the extracts. These findings suggest that among the common constituents, thymol and carvyl acetate may play an active role in DNA damage signaling and apoptosis, thereby contributing to the biological activities of the herb extracts.

Given antioxidant properties associated with these herbs (Figure 1 and Figure 5), we next assessed their antioxidant capacity using the DPPH radical scavenging assay. All three herb extracts exhibited dose-dependent antioxidant activity, with *E. biebersteinii* showing the strongest inhibition (Figure 6C).

To further dissect the contribution of individual compounds, we assessed the antioxidant activity of the four common constituents. Among them, luteolin-7-*O*-rutinoside and thymol displayed measurable radical-scavenging activity. Luteolin-7-*O*-rutinoside produced a modest but significant dose-dependent inhibition (Figure 6D, 2.15% at 12 µg/mL, *p* < 0.0001; 3.17% at 18 µg/mL, *p* < 0.0001). In contrast, thymol exhibited a much stronger antioxidant effect, reaching 18.05% inhibition at 300 µg/mL and 18.08% at 450 µg/mL (*p* < 0.0001 for both). Meanwhile, carvyl acetate and menthyl acetate did not show significant antioxidant activity at tested concentrations (carvyl acetate: max 0.37%, *p* > 0.87; menthyl acetate: max 0.55%, *p* > 0.13), indicating they are unlikely to contribute to the antioxidant effects of the extracts.

## 3. Discussion

### 3.1. Herbal Extracts Induce Germline-Specific DNA Damage Checkpoint Activation and Meiotic Defects in C. elegans

All three herbal extracts—*M. longifolia*, *S. orientalis*, and *E*. *biebersteinii*—exhibited strong nematocidal activity, reducing viability and inducing developmental arrest in *C. elegans*. These phenotypes were accompanied by a significant increase in the high incidence of males (HIM) phenotype, indicative of X chromosome nondisjunction and activation of DNA damage checkpoint and defective DNA repair (Figure 2, Figure 3 and Figure 4). The observed nematocidal activity and the associated HIM phenotype were not attributable to indirect *E. coli*-mediated toxicity, as bacterial growth remained unaffected by extract treatment.

Our multi-layered analysis—linking organism-level phenotypes to cellular, genetic, and molecular markers—demonstrates that these herbal extracts induce germline-specific defects through activation of conserved DNA damage checkpoint pathways. This systems-level approach offers a comprehensive view of the reproductive toxicity caused by botanical mixtures.

### 3.2. Herbal Extracts Lead to Defective Mitotic and Meiotic Progression, Impaired DNA Repair, and DNA Damage Checkpoint Activation, Resulting in Germline Apoptosis

DAPI staining of dissected gonads revealed that *S.o*. and *E.b*. disrupted the spatial organization of germline nuclei. The presence of crescent-shaped nuclei beyond the transition zone, as well as increased nuclear gaps, suggest premature meiotic entry and impaired control of the mitosis-to-meiosis switch. *S.o*. treatment additionally disrupted the premeiotic tip (PMT), pointing to broader developmental dysregulation. These morphological disruptions correlate with reduced germline length and decreased fertility.

All three extracts induced transcriptional upregulation of key DNA damage checkpoint regulators—*atm-1* and *atl-1*—with accompanying increases in pCHK-1 foci and germline apoptosis. These effects were particularly pronounced in *S.o*. and *E.b*.-treated animals. This suggests that the extracts induce genotoxic stress or replication challenges sufficient to activate the DNA damage response, leading to checkpoint-mediated apoptotic removal of compromised germ cells.

Although *M.l*. showed milder phenotypes, it still significantly elevated checkpoint gene expression and pCHK-1 foci, indicating that even low-grade germline stress is sufficient to engage surveillance pathways.

Among the three extracts, *S.o*. produced the most severe phenotypes, including a reduction in diakinesis-stage bivalents, indicative of defective homolog pairing or recombination. Furthermore, RAD-51 foci were significantly elevated in the PMT and pachytene stages following *S. orientalis* treatment, suggesting impaired double-strand break (DSB) repair or the persistence of recombination intermediates. However, we cannot exclude the possibility that this increase reflects the induction of a greater number of DSBs at this stage. These disruptions likely compound DNA damage signaling, culminating in heightened apoptosis.

### 3.3. Phytochemical Composition Underlies the Biological Activities of Herbal Extracts: Four Common Compounds Identified—Thymol, Carvyl Acetate, Luteolin-7-O-Rutinoside, and Menthyl Acetate

LC-MS profiling revealed both shared and species-specific compounds across the three extracts. Notably, four compounds—thymol, carvyl acetate, luteolin-7-*O*-rutinoside, and menthyl acetate—were common to all extracts; of these, thymol and carvyl acetate significantly upregulated *atm-1* and *atl-1* and increased germline apoptosis, effectively recapitulating the effects of the full extracts. In contrast, luteolin-7-*O*-rutinoside and menthyl acetate showed no such activity, underscoring the functional specificity of individual phytochemicals. This finding suggests that a subset of shared compounds may mediate the core genotoxic effects observed across all extracts, while species-specific compounds and the combination of compounds may modulate their severity.

### 3.4. Phytochemical Overlap Explains Parallel DNA Damage Responses Induced by S. orientalis and E. biebersteinii Extracts

We next asked whether the phytochemical similarities between extracts could explain their shared phenotypic profiles. Interestingly, *S.o*. and *E.b*. exhibited the most phenotypic similarity among the three extracts, manifesting nearly indistinguishable effects on germline disorganization, apoptosis, and checkpoint activation. This similarity is supported by their phytochemical profiles: 12 out of 13 major compounds in *S.o*. were also found in *E.b*., suggesting a shared chemical basis for their biological effects.

In addition to the shared compounds, *S.o*. and *E.b*. both contain misoprostol and aucubin, which have been linked to modulation of DNA damage and repair pathways. Misoprostol has demonstrated radioprotective effects in mammalian models by mitigating DNA damage-induced apoptosis [89], while aucubin has been implicated in topoisomerase-mediated DNA repair regulation and has shown therapeutic relevance in cancer settings [90]. These compounds may enhance or synergize with the shared DDR-active constituents to produce stronger germline toxicity.

Moreover, resveratrol, uniquely present in *S.o*., is a well-known polyphenol with multiple pharmacologic activities, including promotion of IR-mediated apoptosis [91,92]. Resveratrol has been shown to sensitize tumor cells to radiation and enhance DNA damage-induced apoptosis, and may contribute to the severity of phenotypes seen in *S.o*.-treated animals.

### 3.5. Uncoupling Antioxidant Activity from Germline Toxicity in Herbal Extracts

While genotoxicity emerged as a major effect of the extracts, we also considered whether antioxidant properties might modulate or counterbalance these effects. All three extracts showed dose-dependent antioxidant activity in DPPH assays, with *E.b.* being the most potent. However, no clear relationship was observed between the genotoxic and apoptotic effects and antioxidant capacity. This disparity may be due to differences in compound bioavailability, metabolism, or the presence of other bioactive constituents that may influence cytotoxic effects. Among shared compounds, thymol contributed to both antioxidant and pro-apoptotic activity, whereas carvyl acetate induced apoptosis without radical-scavenging effects.

These findings indicate that the biological effects of the extracts cannot be explained solely by oxidative stress modulation. Instead, distinct compounds within each extract exert functionally divergent effects—some activating protective antioxidant pathways, others engaging pro-apoptotic DNA damage signaling.

Our findings reveal that the germline phenotypes and fertility defects observed in *C. elegans* upon treatment with *M. longifolia*, *S. orientalis*, and *E. biebersteinii* extracts are the result of both shared and species-specific phytochemicals. Among the four compounds common to all three extracts, thymol and carvyl acetate specifically induced DNA damage checkpoint activation and pachytene-stage apoptosis, while thymol and luteolin-7-*O*-rutinoside contributed to antioxidant activity. The identification of carvyl acetate as a potent apoptosis inducer without antioxidant activity highlights its distinct and potentially toxic function. Meanwhile, species-specific constituents—such as ursolic acid and caryophyllene in *M. longifolia*, or resveratrol in *S. orientalis*—may contribute additional, non-overlapping biological effects.

Importantly, this study illustrates how the interaction between shared and unique compounds drives the complex and divergent biological outcomes of each herbal extract. By establishing a clear correlation between LC-MS-derived chemical profiles and in vivo physiological effects, we provide a mechanistic framework for understanding how multi-component herbal formulations act in biological systems. In particular, we examined the biological effects of four compounds—luteolin-7-*O*-rutinoside, thymol, carvyl acetate, and menthyl acetate—selected for their functional relevance. Notably, all four were commonly identified across the three herbs. Building on these findings, future studies will investigate various combinations of these compounds to explore potential synergistic or antagonistic effects, as part of a broader effort to elucidate the complex bioactivity of the extracts.

## 4. Materials and Methods

### 4.1. Strains and Alleles

*C. elegans* strains were cultured at 20 °C under standard laboratory conditions, following established protocols [93]. The N2 Bristol strain, used as the wild-type control, was obtained from the Caenorhabditis Genetics Center (CGC).

### 4.2. Herb Extraction

Herbal materials were sourced from Armenia and processed as previously reported [33,35]. In summary, plant samples were washed, air-dried, and coarsely ground before undergoing methanol extraction. The resulting methanolic extract was concentrated, reconstituted in 90% aqueous methanol, and partitioned with hexane. The residual hydroalcoholic phase was freed of the solvent in vacuo, suspended in water, and then sequentially extracted with dichloromethane and butanol to afford a gross separation into hexane-, dichloromethane-, butanol-, and water-soluble fractions. Solvent selection was tailored to each plant species: *E. biebersteinii* was extracted using butanol, *M. longifolia* with dichloromethane, and *S. orientalis* with water.

All hexane-based extracts were redissolved in DMSO, standardized to 1 mg/mL, and then diluted in M9 buffer to a working concentration of 0.03 µg/mL for most assays, unless otherwise noted. This concentration was determined based on preliminary dose-dependency tests. In preliminary tests, 0.01% (*v*/*v*) butanol and hexane were applied as solvent controls and showed no observable effects on *C. elegans* survivability or development. DMSO (≤0.1%) was used as the standard vehicle control in all subsequent experiments.

### 4.3. Survival, Larval Arrest/Lethality, and High Incidence of Males (HIM) Assay

Synchronized L1 larvae were prepared by collecting gravid hermaphrodites from NGM plates, using the method described by Kim and Colaiacovo [31,94]. The larvae were then exposed to 180 µL of herbal extract solution in 96-well plates. Following brief agitation, the plates were incubated at 20 °C for 24 h, with phenotypic observations extending up to 48 h. Worm survival was determined based on movement after 24 h of treatment. Brood size was calculated by counting the total number of eggs laid per worm over a 4–5 day period following the L4 stage. Larval arrest or lethality was expressed as the percentage of hatched larvae that failed to reach adulthood. The high incidence of males (HIM) phenotype was assessed by calculating the percentage of males among the adult population. Differences among genotypes were analyzed using the two-tailed Mann–Whitney test, applying a 95% confidence interval (C.I.). Each experiment was independently replicated three times to ensure consistency. This procedure was modified from the protocol established by Kim and Colaiacovo [94].

### 4.4. LC–MS/MS Analysis

Liquid chromatography–tandem mass spectrometry (LC–MS/MS) was carried out according to established protocols [33,35]. Briefly, the analysis was conducted using a Shimadzu LC-30A system equipped with a C18 column, with all procedures performed by YanBo Times (Beijing, China). Compound identification was verified through comparison with a standardized reference database. All detected compounds were authenticated through this stringent methodology. The English names in the LC–MS output were translated from the original Chinese names supplied by YanBo Times.

### 4.5. Immunofluorescence Assay

Whole-mount gonads were stained for immunofluorescence following previously described methods [31,32,95]. The primary antibody used was rabbit anti-phospho-CHK-1 (Ser345) at a 1:250 dilution (Cell Signaling Technology, Danvers, MA, USA), followed by Cy3-conjugated anti-rabbit secondary antibody at a 1:300 dilution (Jackson, Archbold, OH, USA). Fluorescent images were captured using a Nikon Eclipse Ti2-E (Nikon, Tokyo, Japan) inverted microscope paired with a DS-Qi2 camera. Imaging was conducted at 0.2 μm Z-steps using a 60× objective lens with an additional 1.5× magnification. Image processing and deconvolution were performed using Nikon NIS Elements software (Ver 4.3). Figures display either full or partial nuclear projections.

### 4.6. pCHK-1 Foci Quantification

The number of pCHK-1 foci was quantified following established protocols ([31,32]). For each condition, five to ten germlines were examined. Statistical analysis was performed using either a two-tailed Mann–Whitney U test or a standard *t*-test, applying a 95% confidence interval.

### 4.7. Assessment of Germline Apoptosis

Germline apoptosis was evaluated via acridine orange staining in synchronized animals, 20 h after reaching the L4 stage, as previously described [96]. Between 20 and 30 gonads were scored per condition using a Nikon Ti2-E fluorescence microscope. Statistical comparisons were conducted using the two-tailed Mann–Whitney test, with significance set at a 95% confidence level.

### 4.8. qRT-PCR

Total RNA was isolated from young adult hermaphrodites and reverse-transcribed into cDNA using the ABscript II First Strand Synthesis Kit (ABclonal, RK20400, Woburn, MA, USA), as previously described [97,98]. qRT-PCR was carried out using ABclonal 2X SYBR Green Fast Mix (RK21200) on the LineGene 4800 system (BIOER, FQD48A, Hangzhou, China). Thermal cycling conditions included an initial denaturation at 95 °C for 2 min, followed by 40 amplification cycles at 95 °C for 15 s and 60 °C for 20 s, with extension. A melting curve analysis (60–95 °C) was performed to confirm product specificity. The *tba-1* gene, which encodes tubulin, was used as an internal reference, based on previously published *C. elegans* microarray data. All PCR reactions were repeated at least twice to ensure reproducibility.

### 4.9. Chemical Reagents

All reagents used in this study were of analytical grade and were purchased from commercial suppliers, including Sigma-Aldrich, St. Louis, MO, USA. The following chemicals were utilized: hexane (CAS No. 110-54-3), dichloromethane (CAS No. 75-09-2), butanol (CAS No. 71-36-3), dimethyl sulfoxide (DMSO; CAS No. 67-68-5), DAPI (CAS No. 28718-90-3), luteolin-7-*O*-rutinoside (CAS No. 20633-84-5), thymol (CAS No. 89-83-8), carvyl acetate (CAS No. 97-42-7), menthyl acetate (CAS No. 89-48-5), nocodazole (CAS No. 31430-18-9), and acridine orange (CAS No. 65-61-2).

### 4.10. Monitoring the Growth of E. coli

The growth of *E. coli* OP50 in the presence of herb extracts was assessed by measuring its optical density (OD) at 600 nm, following the method described in [35,99]. To determine the antibacterial effects, bacterial growth was monitored using 0.03 µg/mL of each herb extract.

### 4.11. Quantitative Analysis of RAD-51 Foci

Quantitation of RAD-51 foci was performed as described in [95]. RAD-51 foci were quantified in germline nuclei of age-matched hermaphrodites, fixed 24 h post-L4; five and ten germlines were scored for each treatment. Statistical comparisons between treatments were performed using the two-tailed Mann–Whitney or *t*-test with a 95% confidence interval.

### 4.12. DPPH Free Radical Scavenging Assay

The free radical scavenging capacity was determined using DPPH, as described in [100,101]. In brief, a 0.004% DPPH solution was added to achieve a final volume of 3 mL. The mixture was then incubated for 30 min at room temperature before the absorbance was measured at 517 nm.

## Figures and Tables

**Table 1:** Taxonomy, Characteristics, and Distribution of Three Medicinal Herbs.

Genus	Taxonomy	Characteristics	Distribution	Sample Used in This Study
*Mentha*	Defined as 18–30 species across five sections: *Mentha*, *Preslia*, *Audibertia*, *Eriodontes*, *Pulegium*. Includes *M. spicata*, *M. aquatica*, *M. arvensis*, *M. longifolia* [1,2,3,4]	Aromatic, herbaceous perennials with extensive stolons [4]	Widely distributed: Northern Pakistan, Europe, Nepal, India, Western China, Germany, UK, Egypt, Nigeria, Turkey [1]	*Mentha longifolia*
*Scrophularia*	Genus *Scrophularia* (Scrophulariaceae); ~300 species [5]	Mostly herbaceous perennials; also subshrubs, biennials, or annuals [6]	Temperate Asia, Mediterranean Europe, North America [7]	*Scrophularia orientalis*
*Echium*	Genus *Echium* (Boraginaceae); ~60 species, 30 in Canary Islands, 24 endemic [8]	Annual, biennial, or perennial flowering plants [8,9]	Native to North Africa, Europe, Macaronesia (Azores, Madeira, Canaries, Cape Verde) [8,9]	*Echium biebersteinii*

**Table 2:** Reported biological functions of major compounds in three herbal extracts. Each compound—such as antioxidant, DNA damage response/repair, antitumor, and anti-inflammatory functions—is based on previous reports. However, many compounds remain insufficiently characterized and require further investigation. LC-MS spectra of the phytochemicals are provided in Supplementary Datas S3 and S4.

No	Compounds	Antioxidant	DNA Damage Response/Repair	Antitumor	Anti-Inflammatory
1	Luteolin-7-*O*-Rucoside	[36]	[37]	[38]	[39]
2	Thymol	[40]	[41]	[42]	[43]
3	Carvyl acetate				
4	Menthyl Acetate				[44]
5	Luteolin	[45]	[46]	[47]	[48]
6	Caryophyllene	[49]	[50]	[51]	[52]
7	Geranium lignin/Diosmetin	[53]	[54]	[55]	[56]
8	Genistein	[57]	[58]	[59]	[60]
9	Isoquercetin	[61]		[62]	[63]
10	Naringin	[64]	[65]	[66]	
11	Ursolic acid	[67]	[68]	[69]	[70]
12	Phlogistic acid				
13	Dihydrocarvone				
14	Resveratrol	[71]	[72]	[73]	[74]
15	Aucubin	[75]		[76]	[77]
16	Lycopene	[78]	[79]	[80]	[81]
17	Linoleic acid		[82]	[83]	[84]
18	Misoprostol	[85]			
19	Homoplantain				[86]
20	Vitexin-4-*O*-glucoside	[87]			
21	Vitexin-4-rhamnoside	[88]			

## Data Availability

Data is contained in the paper.

## Supplementary Materials

The following supporting information can be downloaded at: https://www.mdpi.com/article/10.3390/ph18071030/s1, Data S1: Traditional Uses of Mentha, Scrophularia, and Echium Species; Data S2: Reported Bioactivities of Genus Mentha, Scrophularia, and Echium; Data S3: LC-MS spectra of phytochemicals identified in *S. orientalis*, *M. longifolia*, and *E. biebersteinii*. The x-axis represents retention time (RT, in minutes), and the y-axis shows relative intensity (%). Each peak corresponds to a phytochemical detected in the respective plant extract. Spectra are provided for both positive (Pos) and negative (Neg) ionization modes for each extract. Analyses were performed using a Shimadzu LC-30A system equipped with a C18 column, with all procedures conducted by YanBo Times; Data S4: Summary of LC-MS analysis of 21 identified compounds from three herb extracts. The table includes each compound’s component name, CAS number, molecular formula, fragment ions (m/z) derived from MS analysis, adduct type, and retention time (RT). For each of the three herbal extracts—*Mentha longifolia* (*M.l.*), *Scrophularia orientalis* (*S.o.*), and *Echium biebersteinii* (*E.b.*)—the peak intensity (%) measured by LC-MS and the corresponding relative quantity (%) (normalized to the most abundant compound in each extract, set as 100%) are shown. References [102,103,104,105,106,107,108,109,110,111,112,113,114,115,116,117,118,119,120,121,122,123,124,125,126,127,128,129,130,131,132,133,134,135,136,137,138,139,140,141,142,143,144,145,146,147,148,149,150,151,152,153,154,155,156,157,158,159,160,161,162,163,164,165,166,167,168,169,170,171,172,173,174,175,176,177,178,179,180,181,182,183] cited in Supplementary Materials.
