# Supplementary material for: Herbal Extract-Induced DNA Damage, Apoptosis, and Antioxidant Effects of C. elegans: A Comparative Study of Mentha longifolia, Scrophularia orientalis, and Echium biebersteinii
Source: Pharmaceuticals (Basel). 2025 Jul 11;18(7):1030. doi: 10.3390/ph18071030 (PMC12299367; doi:10.3390/ph18071030)
Supplement: Supplementary file 1 [file pharmaceuticals-18-01030-s001.zip › pharmaceuticals-3676912-supplementary.pdf]

Supplementary Data

Supplementary data S1. Traditional Uses of Mentha, Scrophularia, and Echium Species

Genus	Traditional Use
Mentha	*Mentha longifolia* has been traditionally used for treating gastrointestinal, respiratory, infectious, inflammatory, and menstrual disorders across various cultures worldwide [24].
Scrophularia	*Scrophularia ningpoensis* is a traditional medicinal plant in China used for treating fever, swelling, constipation, pharyngitis, neuritis, and laryngitis. Various iridoid glycosides and related compounds have been isolated from it [102-104].
Echium	*Echium amoenum* was historically used by Romans for colds and fevers (via decoctions), and mixed with wine for mood enhancement [9]. *Echium italicum* is a traditional Turkish remedy for wound healing, abscesses, rheumatic pain, and hemorrhages. Used as poultice or ointment [9, 105].

Supplementary data S2. Reported Bioactivities of Genus Mentha, Scrophularia, and Echium

Genus	Anti-proliferative	Anti-inflammatory	Antioxidant	Anti-bacterial
**Mentha**	Crude extract of *M. longifolia* inhibited viability of adrenocortical tumor cells (SW13, H295R); strongest at >0.5 μg/μl after 72 h. Mitotane combination showed no added efficacy [28].	Hexane extract of *M. longifolia* reduced NO production and suppressed iNOS and TNF-α in LPS-stimulated macrophages. Aqueous extract also exhibited antipyretic and antinociceptive effects [106, 107].	Essential oil and extracts showed DPPH scavenging, inhibition of linoleic acid oxidation, and hydroxyl radical neutralization [27].	Menthol from *M. longifolia* exhibited antibacterial effects (e.g., *S. aureus*, *S. pyogenes*) and antifungal activity (*Candida albicans*). Flavones may also inhibit HIV in vitro [25, 26].
References	[108-110]	[111-117]	[118-120]	[115, 121-125]
**Scrophularia**	*S. orientalis*, *S. striata*, *S. floribunda*, and *S. lucida* extracts reduced viability of cancer cell lines via necrosis or apoptosis[12-14].	*S. ningpoensis* extract reduced cytokine levels in inflammation models. *S. megalantha* modulated immune cytokine profiles (↑IL-10, ↓IL-17, IFN-γ) [126, 127].	Phenylpropanoid glycosides (PGs) from *S. ningpoensis* reduced oxidative DNA damage [128].	Extracts from *S. deserti*, *S. frutescens*, *S. sambucifolia*, and *S. striata* exhibited antibacterial effects, including against *B. melitensis* and resistant *P. aeruginosa* [129-131].
References	[132-134]	[135-143]	[135, 143-148]	[149-157]
**Echium**	*E. amoenum* and its compound rosmarinic acid suppressed gastric cancer cell growth via apoptosis and inhibition of STAT3, AKT, and ERK1/2 pathways [22].	*E. amoenum* hexane extract reduced NO, iNOS, COX2, IL-1β, IL-6, TNF-α in macrophages [158]	Decoction and hydroalcoholic extracts of *E. amoenum* showed high antioxidant activity due to rosmarinic acid content [159].	*E. amoenum* extract inhibited viruses (e.g., bacteriophage 3C) and pathogens (*S. aureus*). *E. altissimum* methanol extract showed antibacterial activity against *M. luteus* [160-162].
References	[163-165]	[166-170]	[17, 171-176]	[15, 177-183]

***M.l* (Neg)**

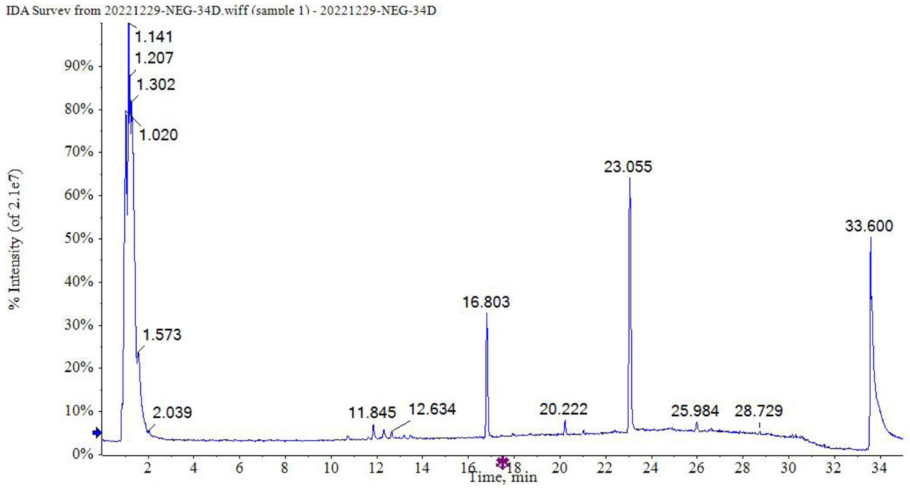

***M.l* (Pos)**

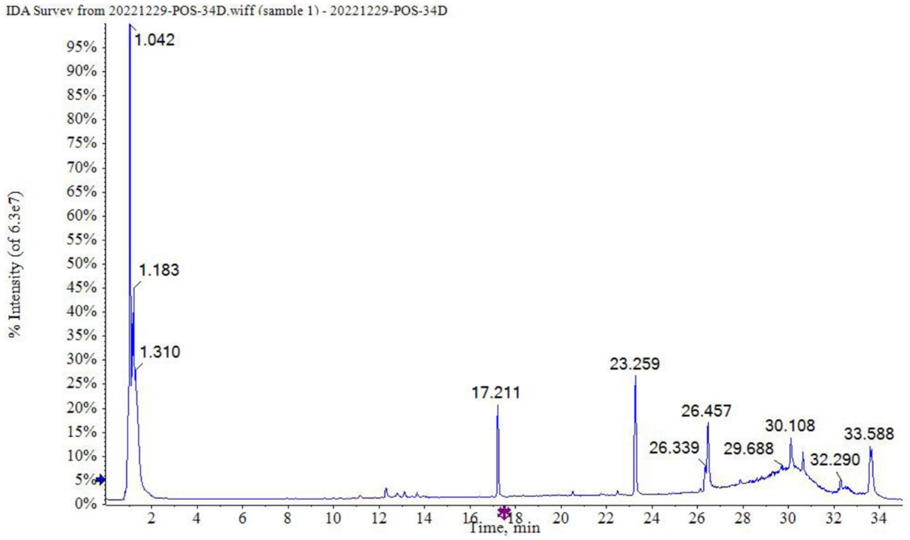

***S.o* (Neg)**

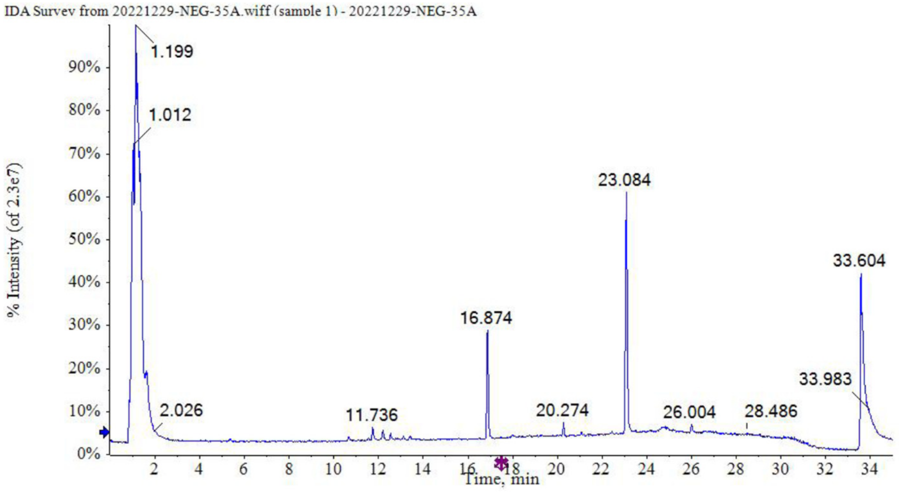

***S.o* (Pos)**

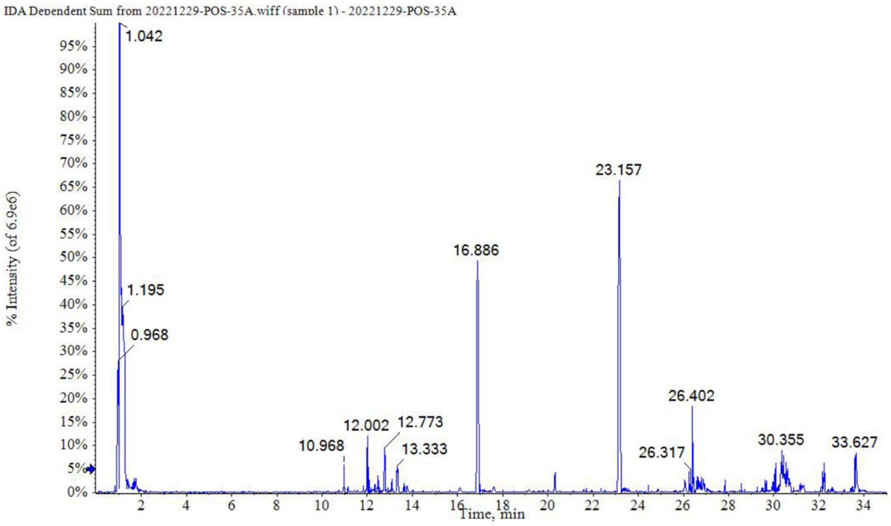

***E.b* (Neg)**

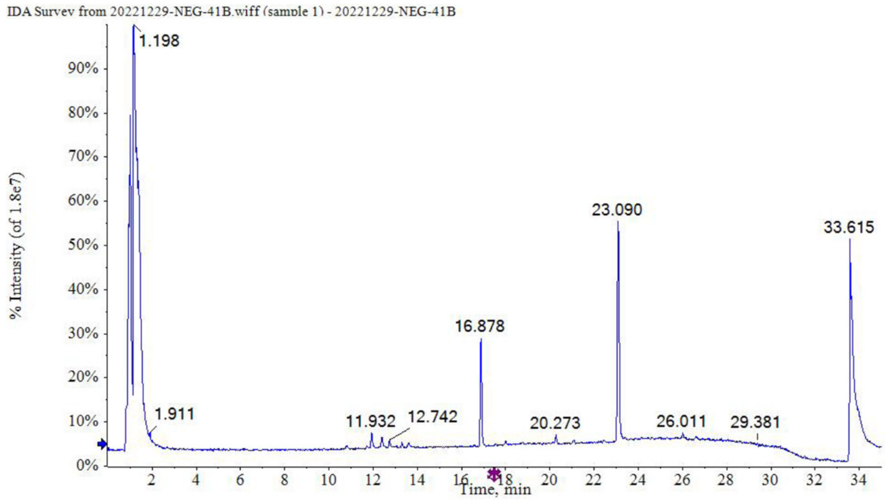

***E.b* (Pos)**

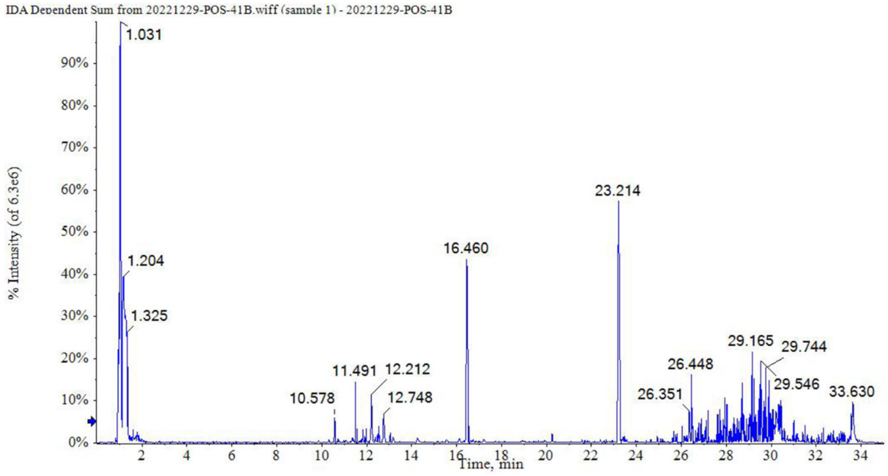

Supplementary Data S3. LC-MS spectra of phytochemicals identified in *S. orientalis*, *M. longifolia*, and *E. biebersteinii*. The x-axis represents retention time (RT, in minutes), and the y-axis shows relative intensity (%). Each peak corresponds to a phytochemical detected in the respective plant extract. Spectra are provided for both positive (Pos) and negative (Neg) ionization modes for each extract. Analyses were performed using a Shimadzu LC-30A system equipped with a C18 column, with all procedures conducted by YanBo Times.

No	Component	CAS Number	Formula	Fragment ions (m/z)	Adduct	RT	Intensity(%) in *M.l.*	Relative Quantity (%) in *M.l.*	Intensity(%) in *S.o.*	Relative Quantity (%) in S.o.	Intensity(%) in *E.b.*	Relative Quantity (%) in *E.b.*
1	Luteolin-7-O-Rucoside	20633-84-5	595.1654	286.0476、301.0713、463.1228	+H	12.105	3	10.3	5	9.1	1.5	3.3
2	Thymol	89-83-8	151.1119	81.0734、91.0567、105.0705、107.0865	+H	20.681	2	6.9	4	7.3	2	4.4
3	Carvyl acetate	1205-42-1	195.1374	153.1284	+H	17.201	20.5	70.7	2	3.6	45	100
4	Menthyl Acetate	89-48-5	199.1692	155.0525、128.0622、69.0750	+H	27.946	5	17.2	3	5.5	3.8	8.4
5	Luteolin	491-70-3	287.0549	168.0056、140.0109	+H	1.308	29	100	32	58.2		
6	Caryophyllene	87-44-5	205.1952	149.0243、135.1168	+H	27.288	4	13.8				
7	Geranium lignin/Diosmetin	520-34-3	301.0707	286.0493、285.0395、168.0054、140.0108	+H	12.789	2.5	8.6				
8	Genistein	2798-20-1	359.1128	162.0687、211.0736、270.0860、298.0843、326.0798	+H	21.632	2	6.9				
9	Isoquercetin	21637-25-2	465.1018	287.0565	+H	1.531	6	20.7				
10	Naringin	10236-47-2	581.1865	455.7599、273.0780、263.0545、153.0191	+H	13.317	1.5	5.2				
11	Ursolic acid	77-52-1	457.3679	411.3622、297.2594、191.1804	+H	29.232	7	24.1				
12	Phlogistic acid	501-97-3	167.0703	165.0681、149.0606、149.0276、121.0317、95.0511、77.0421	+H	23.13	24	82.8				
13	Dihydrocarvone	5524-05-0	153.1273	152..1122	+H	26.745			3	5.5	10	22.2
14	Resveratrol	501-36-0	229.085	151.0381	+H	23.621			2	3.6		
15	Aucubin	479-98-1	347.1335	216.9992	+H	26.495			11	20	4.3	9.6
16	Lycopene	502-65-8	315.0791	257.0749、182.0428	+H	1.104			55	100	32	71.1
17	Linoleic acid	60-33-3	281.2466	265.0143、151.0293	+H	23.793			1	1.8	1.3	2.9
18	Misoprostol	848849-83-2	287.2361	173.1341、159.1143	+H	27.492			0.5	0.9	7	15.6
19	Homoplantain	17680-84-1	463.1221	301.0705、286.0470	+H	12.49			4	7.3	4	8.9
20	Vitexin-4-O-glucoside	76135-82-5	595.1637	301.0709、286.0474	+H	11.846			1	1.8	1.5	3.3
21	Vitexin-4-rhamnoside	32426-34-9	579.1703	301.0709、286.0474	+H	12.126					4	8.9

Supplementary Data S4. Summary of LC-MS analysis of 21 identified compounds from three herb extracts. The table includes each compound's component name, CAS number, molecular formula, fragment ions (m/z) derived from MS analysis, adduct type, and retention time (RT). For each of the three herbal extracts — *Mentha longifolia (M.l.)*, *Scrophularia orientalis (S.o.)*, and *Echium biebersteinii (E.b.)* — the peak intensity (%) measured by LC-MS and the corresponding relative quantity (%) (normalized to the most abundant compound in each extract, set as 100%) are shown.
